# Temperature increase and fluctuation induce phytoplankton biodiversity loss – Evidence from a multi‐seasonal mesocosm experiment

**DOI:** 10.1002/ece3.2889

**Published:** 2017-03-22

**Authors:** Serena Rasconi, Katharina Winter, Martin J. Kainz

**Affiliations:** ^1^ Inter‐university Center for Aquatic Ecosystem ResearchWasserCluster Lunz – Biologische StationLunz am SeeAustria

**Keywords:** aquatic plant ecology, climate change, community structure, Cyanobacteria dominance, food web

## Abstract

Global climate change scenarios predict lake water temperatures to increase up to 4°C and extreme weather events, including heat waves and large temperature fluctuations, to occur more frequently. Such changes may result in a reorganization of the plankton community structure, causing shifts in diversity and structure toward a community dominated by fewer species that are more adapted to endure warmer and irregular temperature conditions. We designed a long‐term (8 months) mesocosm experiment to explore how ambient water temperature (*C*: control), induced increased temperature (*T*: +4°C), and temperature fluctuations (*F*: ±4°C relative to *T*) change phytoplankton phenology, taxonomical diversity, and community structure, and how such changes affected zooplankton abundance and composition. *Synthesis*. Our results show that *T* and *F* relative to *C* significantly decreased phytoplankton diversity. Moreover, there was a clear effect of the temperature treatments (*T* and *F*) on phytoplankton size structure that resulted in a significantly lower growth of large species (i.e., large Chlorophyta) compared to *C*. Decreased diversity and evenness in the *T* and *F* treatments pushed the community toward the dominance of only a few phytoplankton taxa (mainly Cyanobacteria and Chlorophyta) that are better adapted to endure warmer and more irregular temperature conditions. The observed shift toward Cyanobacteria dominance may affect trophic energy transfer along the aquatic food web.

## 1 Introduction

As a result of global warming, lake temperatures are predicted to increase by up to 4°C, together with an increase in variability of weather events. In addition to the warmest temperatures recorded during the past 500 years (Luterbacher, Dietrich, Xoplaki, Grosjean, & Wanner, 2004), extreme weather events, such as heat waves or flood, are expected to increase in length, frequency, and/or intensity (Field, 2012). For aquatic ecosystems, temperature is one of the most important environmental factors that affect the growth of primary producers (Eppley, 1972) and planktonic communities (Graham & Vinebrooke, 2009). It can be expected that increasing temperature will lead to increasing phytoplankton growth rates, nutrient uptake, and overall metabolic activity (Litchman, Klausmeier, Schofield, & Falkowski, 2007) with a consequent switch of the community toward being dominated by species with high turnover rates and short‐standing biomass (Yvon‐Durocher, Jones, Trimmer, Woodward, & Montoya, 2010). Qualitative and quantitative changes at the base of aquatic food webs may subsequently alter the trophic energy transfer conveyed to consumers (Behrenfeld, 2014) and thus potentially affect the entire food chain.

Decreasing plankton diversity is one of the most evident effects of global warming (Thomas, Kremer, Klausmeier, & Litchman, 2012). A short‐term heat stress (i.e., +10°C for 1 week) can cause the diversity of marine benthic microalgae to shift toward the dominance of warm‐temperature‐tolerant species (Eggers, Eriksson, & Matthiessen, 2012). In lakes, Konopka & Brock (1978) reported that warmer temperatures favored Cyanobacteria growth due to their photosynthesis optimum at high temperatures and warmer temperatures may extend the Cyanobacteria optimal growth periods (Paerl & Huisman, 2008). Studies conducted in Europe during the summer 2003, one of the hottest recorded during the last century, showed that heat waves directly promoted Cyanobacteria blooms (Joehnk et al. 2008) and temperature fluctuation was found as one of the predictors for Cyanobacteria occurrence (Zhang et al., 2016). In addition to growth rates, temperature may also mediate nutrient availability, which in turn is also an important factor driving phytoplankton growth and diversity (Elser et al., 2007). Warmer waters may increase nutrient uptake that in some lakes caused nitrogen limitation (Elliott, 2012) to the advantage of some nitrogen‐fixing Cyanobacteria (Moisander, Steppe, Hall, Kuparinen, & Paerl, 2003). Moreover, warmer temperature can mediate higher P release from the sediment and promote the dominance of nitrogen‐fixing algal species (most notably dinophytes and Cyanobacteria) (Jeppesen et al., 2009). Warmer temperatures and environmental instability are thus further expected to cause changes in phytoplankton diversity and community structure, which are essential characteristics underlying ecosystem functioning and trophic transfer.

Reduced diversity and dominance of bloom‐forming species including Cyanobacteria may also alter dietary availability and nutritional quality for consumers. Recent experimental evidence suggests that temperature effects on grazers seem to be mostly compromised by resource availability and its biochemical composition (Verbitsky & Verbitskaya, 2011). For example, the filter feeding *Daphnia* decreased the magnitude of spring peak in response to changes in algal composition (Winder et al., 2012) and reduced algal carrying capacity (Schalau, Rinke, Straile, & Peeters, 2008). As warming provoked shifts to smaller plankton species (Rasconi, Gall, Winter, & Kainz, 2015; Yvon‐Durocher, Montoya, Trimmer, & Woodward, 2011), this may change food quality available for consumers at higher trophic levels as pico‐ and nanophytoplankton species are less efficiently assimilated by copepods (Sommer & Sommer, 2006) and often constitute lower nutritional quality for zooplankton (e.g., Cyanobacteria; Elert, Martin‐Creuzburg, & Le Coz, 2003). Indeed, in warming lakes, decreasing zooplankton biomass and zooplankton/phytoplankton biomass ratios were reported (Jeppesen et al., 2009).

In spite of increasing evidence of rapidly warming lakes and ponds (e.g., Graham & Vinebrooke, 2009; O'Reilly et al., 2015), direct temperature effects on phytoplankton diversity and community structure remain poorly understood and difficult to disentangle. In particular, long‐term effects of temperature changes on phytoplankton community diversity and structure clearly warrant further attention. It becomes increasingly clear that biodiversity declines more rapidly in recent years (Allan et al., 2013) and that such effects may vary seasonally (Kratina, Greig, Thompson, Carvalho‐Pereira, & Shurin, 2012). It is thus important to conduct long‐term and multi‐seasonal studies to more precisely assess how aquatic communities respond to altered temperature scenarios.

In an effort to understand how increased temperature and, concurrently, rapidly changing weather events affect phytoplankton diversity and community structure, we designed a long‐term and multi‐seasonal (8 months) mesocosm experiment to test the effects of temperature increase and fluctuation on (1) phytoplankton phenology, taxonomical diversity, and community structure (“primary producer effect”) and (2) how these changes affect zooplankton abundance and composition (“consumer effect”). We expected that increased temperature would cause plankton community composition to shift toward smaller‐sized species that are more adapted to warmer temperatures (see also Rasconi et al., 2015). Induced temperature fluctuations are further expected to cause changes in phytoplankton diversity by favoring fast‐growing species that are more adapted to rapidly changing environments. This study will provide experimental evidence whether and how temperature changes may alter phytoplankton diversity and push the system toward the dominance of monospecific bloom, thus resulting in altered trophic transfer of dietary nutrients to consumers (Brett et al., 2006; Müller‐Navarra et al., 2004; Taipale et al., 2013) and monopolizing ecosystem functions by only one or very few taxa (Sala & Knowlton, 2006).

## 2 Methods

### 2.1 Experimental setup

Twenty‐four thermally insulated cylindrical polyethylene containers (74 cm diameter × 102 cm height) were placed outside the research center WasserCluster Lunz (47°51′N, 15°01′E) and each filled with and kept at 400 L of surface lake water from nearby Lake Lunz. Lake zooplankton was collected using a zooplankton net (100 μm mesh size), pooled in a bucket, and subsequently equally distributed to each of the mesocosms. Collecting permits were provided through an agreement between the owner of Lake Lunz and WasserCluster Lunz. None of the species collected are considered threatened. This multi‐seasonal mesocosm experiment consisted of three treatments (replicated eight times): (1) a control treatment (“*C*,” ambient temperature), (2) an elevated temperature treatment (“*T*,” +4°C above control temperatures), and (3) a temperature fluctuation treatment (“*F*,” with water temperatures fluctuating ±4°C relative to temperature treatment every 4 weeks, with the same total amount of energy applied as for *T*). Water temperature of all mesocosms was controlled by a computerized system (Hansson et al., 2013). The experiment lasted from October 2014 to May 2015.

Each of the mesocosms was protected from external input of particles by a nylon mesh at the top and was permanently and equally aerated by air diffusers to promote slight air‐induced mixing. As in other studies, the enclosure walls were regularly cleaned to minimize the growth of periphytic algae (see Hansson et al., 2013), which settled to the bottom. Nutrient (P and N) concentrations were measured weekly, and throughout this experiment, all mesocosms were fertilized weekly and equally with the same nutrient input according to the Redfield ratio (3 μg P from K_2_PO_4_ and 45 μg N from NaNO_3_) to avoid nutrient depletion. The nutrient concentrations were kept low to simulate how temperature changes affect oligo‐mesotrophic aquatic ecosystems.

### 2.2 Samples analysis

**Figure 1:**
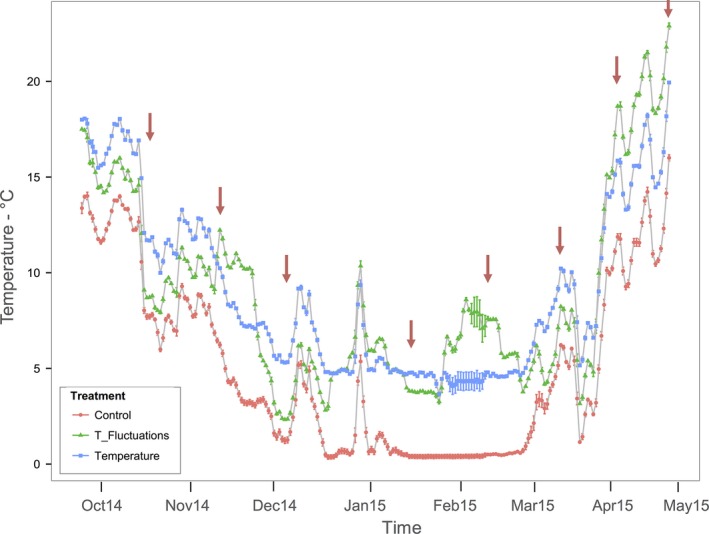
Temperature trend and standard error for all the treatment during the experiment. Samplings are indicated by red arrows

Samples were taken every month at the maximum of temperature difference of *F* from *T* (Figure 1). Integrated samples were taken from each mesocosm using a plastic tube (100 cm length, 6 cm diameter, ~3 L volume) and analyzed the same day. NO_2_‐N, NO_3_‐N, and NH_4_‐N were analyzed using a continuous flow analyzer (FlowSys, Systea). Total phosphorus (TP) was quantified after persulfate digestion followed by molybdate reaction (Wetzel & Likens, 1991), and soluble reactive phosphorus (SRP) was quantified after filtration of acid‐washed filters (Whatman^™^ GF/F). TP and SRP were subsequently analyzed following a molybdate reaction (Wetzel & Likens, 1991) at 880 nm wavelength using a UV/VIS spectrophotometer (UV‐1700). Dissolved organic carbon (DOC) was measured after filtration on precombusted GF/F filters using a TOC analyzer (Sievers 900, GE).

Zooplankton were collected by sieving water (10 L) through a mesh (100 μm) and counted on a stereo‐microscope (Bresser microscope, Germany) at 40× magnification. Phytoplankton (<100 μm) were fixed with Lugol and variable volumes (5–50 ml) were settled following the Utermöhl method (Utermöhl, 1958). Samples were counted on an inverted microscope (Leica DMI 3000 B), and at least 400 cells were identified to the genus level. Phytoplankton biovolumes were assigned using reference data (Kremer, Gillette, Rudstam, Brettum, & Ptacnik, 2014). Heterotrophic bacteria and autotrophic picoplankton were fixed with formaldehyde, stained with Sytox Green, counted in triplicates using a Gallios flow cytometer (Beckman Coulter), and subsequently analyzed using the Kaluza software. Samples were counted under blue laser excitation (488 nm), and based on the autofluorescence of the pigments, it was possible to differentiate autotrophic picoplankton as picochlorophyta (chlorophyll *a* emission at 695 nm) and picocyanobacteria (phycoerythrin emission at 520–620 nm).

Phytoplankton size distribution was separated into two different size classes: small (0–20 μm) and large (20–100 μm cell length). As a proxy for phytoplankton diversity, we used the biovolume repartition among genera. We calculated as diversity indices the genus richness (S, number of genera), the alpha diversity, and the evenness (J) using the R package “Vegan.” For zooplankton, we used the counts of individuals identified at the genus level.

As a measure of trophic transfer efficiency, we calculated the zooplankton/phytoplankton biomass. Seston (<30 μm) was collected on GF/F filters (~500 ml) as the mostly ingestible phytoplankton fraction by zooplankton; seston was freeze‐dried, weighed, and converted as biomass using the Strickland (Strickland, 1966) conversion factor. Zooplankton were freeze‐dried and weighed and the dry weight converted to biomass using Kiørboe (Kiørboe, 2013) conversion factor.

### 2.3 Statistical analysis

Data were analyzed using R (http://www.r-project.org), including the following packages: “DoBy” for data formatting, “lme4” for linear mixed models computation, “profileR” for profile analysis, and “Vegan” for multivariate statistics.

We used nonmetric multi‐dimensional scaling (NMDS) (R Vegan package) to investigate the effect of the treatments in determining the phytoplankton biovolume repartition and to explore the effect of the environmental variables determined by treatments on the identified repartition. For the most represented phytoplankton taxa (Cyanobacteria and Chlorophyta), logistic regression analysis was used to investigate the dominance of phytoplankton community (relative biovolume >70% of the total). We tested the significance of the dominance attributable to the two different taxa and the effects of the treatments (warming and heat waves) using ANOVA.

We fitted linear mixed models using temperature and temperature fluctuations as the difference of temperature in *F* relative to ambient water temperatures (∆_Temp) and TP as fixed‐effects model; as a random effect, we used sampling time (as number of days of the experiment for time effect) and mesocosm replicates. Dependent variables were plankton population abundances (heterotrophic bacteria, phytoplankton and zooplankton), phytoplankton biovolume, Cyanobacteria volume, and dominance (relative biovolume >70% of the total), phytoplankton diversity (number of genera—S, evenness—J, and alpha index diversity), and phytoplankton distribution of the two different size classes mentioned above (small and large). ANOVA was consequently used to test for the best‐fitting model.

We performed profile analysis as repeated measures of variance to identify criterion‐related patterns (diversity indices and phytoplankton biovolume) based on the variation among the treatments (i.e., the levels in the score profile) of the tested variable. To strengthen our results, we ran parallel analysis by groups, that is, one group including all the data from each replicate of the different treatments over time (8 replicates × 8 months = 64 points per treatment).

We used a logistic regression to investigate the effect of the treatments and other environmental parameters (SRP, TP, NO_2_, NO_3_, and NH_4_) on the dominance of one phytoplankton taxon on the entire community. As dichotomous function, we set the Cyanobacteria relative volume with the threshold as relative biovolume >70% (CyanoDom >70%).

The entire dataset was analyzed (192 numbers of observations) and data were log transformed prior to statistical analysis. The statistically significant difference value was set at *p* < .05.

## 3 Results

### 3.1 Physicochemical parameters

Water temperatures were continuously kept +4°C higher in the heated treatment (*T*) relative to the control treatment (*C*; Figure 1). Temperature in the temperature fluctuations treatment (*F*) was fluctuating relative to *T* (Figure 1) from −3.5°C reached on October 2014 to a maximum of +7.73°C reached on February 2015 and relative to *C* from +0.27°C to +8.7°C (Figure S1). The temperatures were significantly different among the treatments (ANOVA; *df* = 2, *F* = 14.4, *p* < .001), and the whole dataset, including the seasonal variations, ranged from 0.01°C to 23.5°C (Data S1), whereby the same amount of energy was supplied to *T* and *F*. Concentrations of SRP ranged from 0 to 14.2 μg/L and TP from 2.4 to 83 μg/L, thus covering a wide seasonal range from oligo‐ to eutrophic status in all treatments. SRP and TP concentrations (μg/L) were significantly different among the treatments during the experiment despite equal fertilization in all the treatments (average SRP ± *SD*:* C* = 0.95 ± 0.99, *T* = 1.18 ± 1.47, *F* = 1.88 ± 2.45, *df* = 2, *F* = 14.4, *p* < .01; average TP ± *SD*:* C* = 14.89 ± 7.55, *T* = 24.41 ± 14.86, *F* = 18.21 ± 14.26, *df* = 2, *F* = 9.08, *p* < .001). Ammonium concentrations (1.8–449.8 μg/L) were higher than nitrite concentrations (1–16.3 μg/L), which were not significantly different in the treatments compared to *C* (*df* = 2, *F* = 3, *p* > .05; ANOVA). Dissolved organic carbon (DOC) concentrations ranged from 6.1 to 22.9 μg/L and were significantly higher in *T* and *F* than in *C* (*df* = 2, *F* = 9.4, *p* ≤ .001; ANOVA).

### 3.2 Plankton phenology

**Figure 2:**
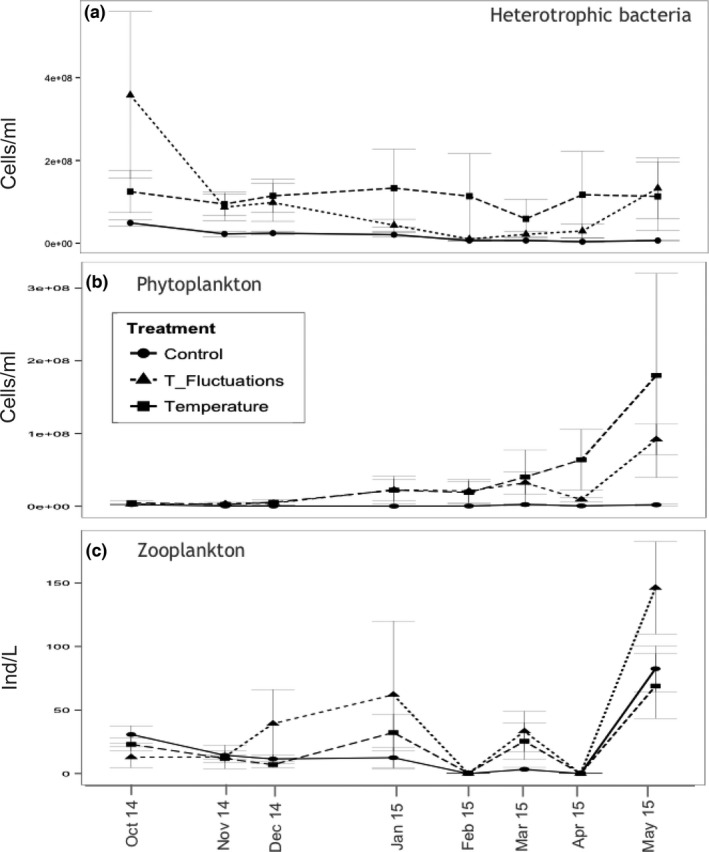
Plankton phenology. Abundance and standard deviations for (a) bacteria, (b) phytoplankton, and (c) zooplankton over time

The abundance of heterotrophic bacteria (Figure 2a) decreased throughout the experiment from initially an average of 3.57 × 10^8^ cells/L (October 2014) to 3.81 × 10^7^ cells/L (May 2015). Highest bacteria abundance was recorded in the *F* treatment in October 2014 (15.91 × 10^8^ cells/L). Average bacteria abundance was highest in *T* (10.8 × 10^8^ cells/L) and higher in *F* (0.97 × 10^8^ cells/L) compared to *C* (0.75 × 10^8^ cells/L) and significantly higher in both temperature treatments (*T* and *F*) compared to *C* (*df* = 2, *F* = 4.96, *p* ≤ .01; ANOVA).

The average abundance of phytoplankton, including picocyanobacteria and picochlorophyta (Figure 2b), ranged from 2.38 × 10^8^ to 179.8 × 10^9^ cells/L and was highest in *T* (4.23 × 10^8^ cells/L), lower in *F* (2.35 × 10^8^ cells/L), and lowest in *C* (1.16 × 10^7^ cells/L). Only as of January 2015, the phytoplankton abundance increased in *T* and *F*, while it decreased in *C*. Phytoplankton abundance was significantly higher in the elevated temperature treatment (*T*) compared to *C* (*df* = 2, *F* = 3.16, *p* < .05; ANOVA).

Zooplankton abundance (Figure 2c) ranged from <1 individuals/L to 146 ind./L. In general, zooplankton abundance was lower at the beginning of the experiment, except for low values recorded in late winter and early spring (February and April 2015). Zooplankton abundance was higher in *F* (max 146–468 ind./L) compared to *T* (max 69–200 ind./L) and lowest in *C* (max 68–179 ind./L) (Figure 2c), but not significantly different (*df* = 2, *F* = 2.159, *p* > .05; ANOVA).

The best‐fitting model for plankton dynamic included temperature and TP as fixed variables, and sampling time (Julian day) and mesocosm replicates as random effects. Model analysis of variance (ANOVA) revealed significant effects of temperature (*df* = 166; *F* = 15.2, and 51.4; *p* ≤ .0001, for phytoplankton abundance and zooplankton abundance, respectively) and TP (*df* = 166; *F* = 60.19 6.1 and 11.9; *p* ≤ .01, for heterotrophic bacteria abundance, phytoplankton abundance, and zooplankton abundance, respectively; Table 1).

**Table 1:** Results of the best‐fit linear mixed‐effect model for plankton population abundances, phytoplankton diversity indices, and phytoplankton community structure

	*df*	AIC	Temp		TP		Δ_Temp	
			*F* ratio	*p* Value	*F* ratio	*p* Value	*F* ratio	*p* Value
Bact.Ab	166	5070	3.5	ns	60.19	<.0001	–	–
Phyto.Ab	166	7481	15.2	.0001	6.1	.014	–	–
Zoo.Ab	166	2039	51.4	<.0001	11.9	<.001	–	–
Div‐S	166	1034	0.45	ns	–	–	10.6	.0014
Div‐J	166	−37.8	0	ns	–	–	9.9	.0019
Div‐alpha	166	94.1	4.1	.04	–	–	10.7	.0013
Small.Biovol	166	7481	15.2	.0001	6.1	.01	–	–
Cyano.Biovol	167	7154	17.3	<.0001	–	–	4.05	.04
CyanoDom	1	213.4	0.4	.01	–	–	10.1	.002

*df*, degree of freedom; AIC, Akaike information criterion; Temp, temperature; TP, total phosphorous; **Δ**_Temp,  temperature difference relative to the temperature treatment.

### 3.3 Phytoplankton and zooplankton taxonomy

**Figure 3:**
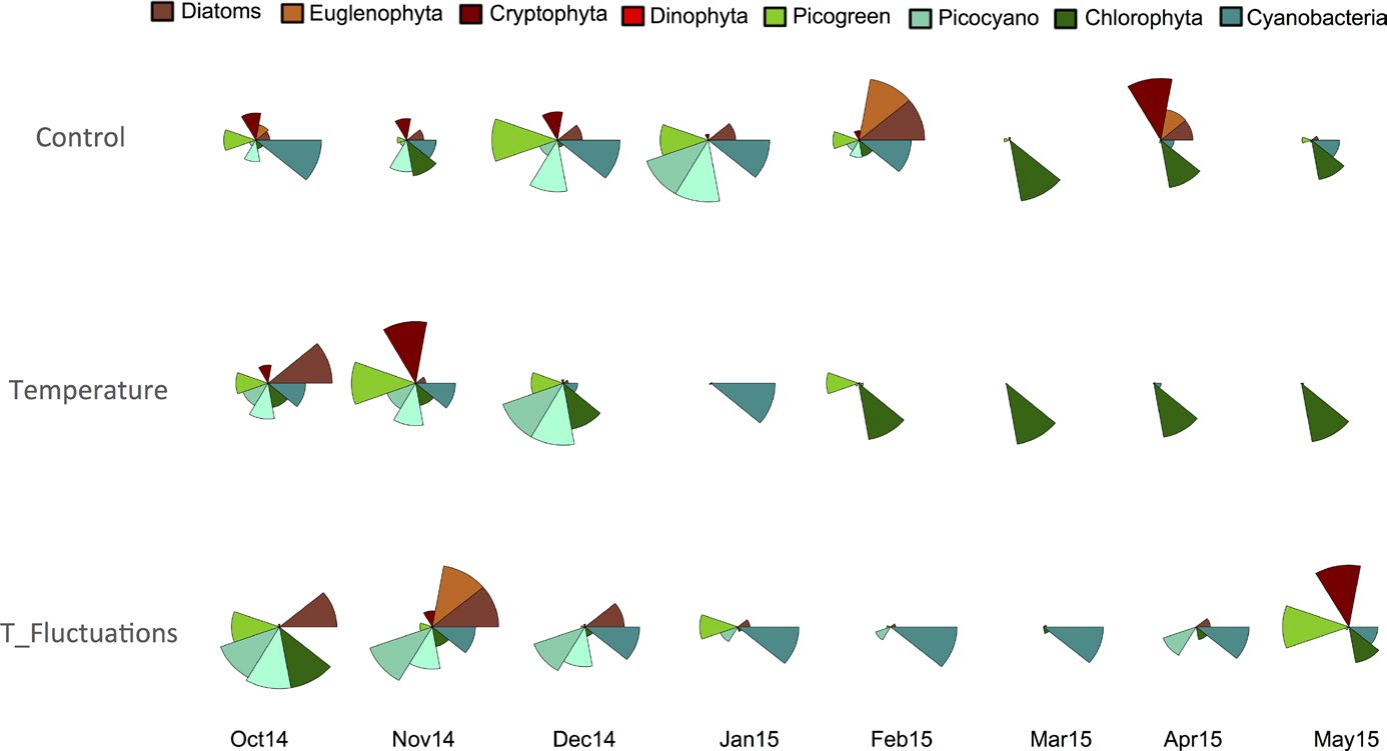
Phytoplankton taxonomic composition (relative biovolume) in each treatment

Phytoplankton diversity was represented mainly by Chlorophyta and Cyanobacteria (Figure 3) throughout the experiment. In C, the most abundant species were the Chlorophyta *Cosmarium*,* Oocystis*,* Scenedesmus*, and *Pandorina*, together with the Dinophyta *Gymnodinium*, and traces of diatoms and Cryptophyta. In T, the most abundant species were *Monoraphidium*,* Crucigeniella*, and *Chlorococcales* among the Chlorophyta, together with the Cyanobacteria *Aphanoteche*,* Chroococcus*, and *Cylindrospermum*. In F, the most represented species were detected within Cyanobacteria, including *Aphanoteche*,* Aphanocapsa*, and *Chroococcus* (Figure 3).

**Figure 4:**
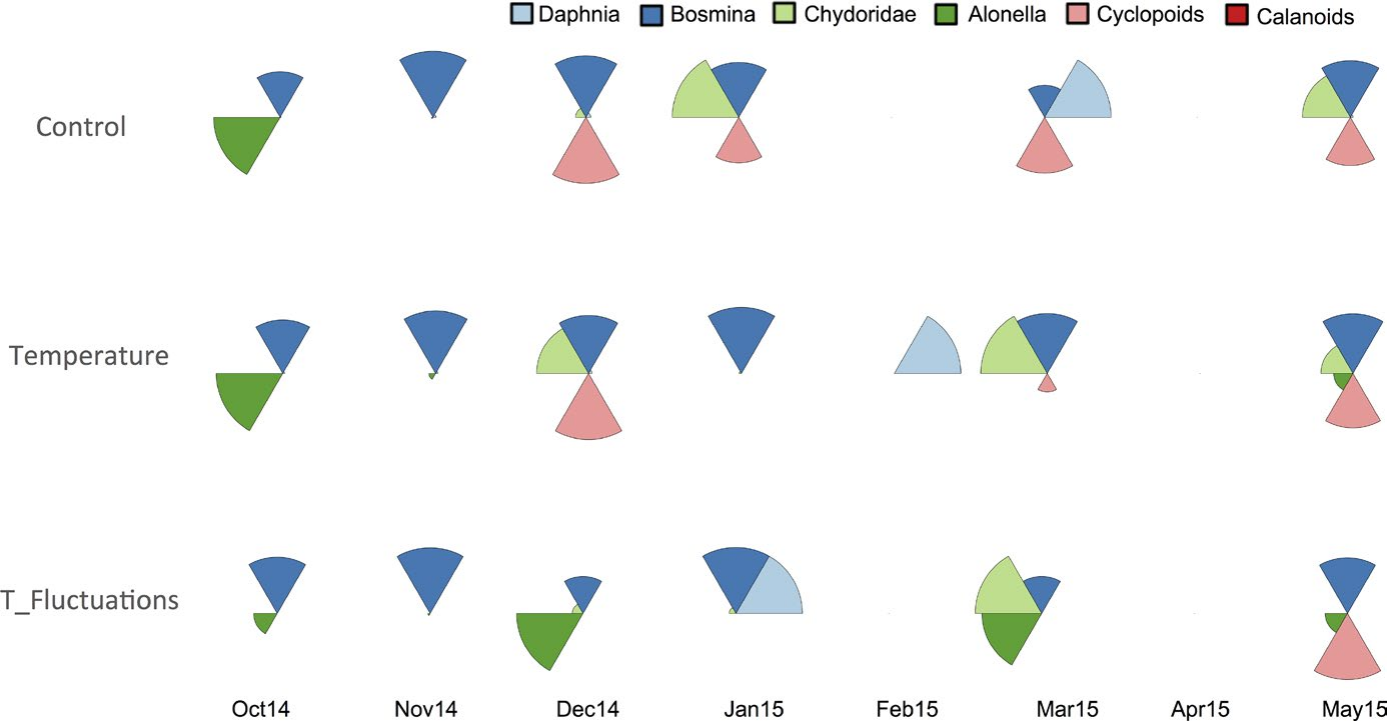
Zooplankton taxonomic composition (relative abundance) in each treatment

Zooplankton was predominantly represented by *Bosmina longirostris* in all treatments throughout the experiment. In October 2014, *Alonella* was recorded in all treatments and proliferated in F, but was still less abundant than *Bosmina*. In C, few Chydoridae were detected during spring 2015 (Figure 4).

### 3.4 Phytoplankton diversity

We observed a continuous decline of phytoplankton richness (number of genera, S) during the experiment. The number of genera was significantly lower in both temperature treatments (*T* and *F*) compared to *C* (*df* = 1, *F* = 68.85, *p* < .0001, ANOVA). The average number of genera was significantly lower in *T* (10.3 genera) and *F* (10.9 genera) compared to *C* (13.2 genera) (*df* = 2, *F* = 19.1, *p* < .0001, ANOVA). Similarly, species repartition (alpha diversity and evenness, J) declined continuously over time and significantly in both temperature treatments (*T* and *F*) compared to *C* (*df* = 1, *F* = 23.89, *p* < .01, ANOVA). Particularly average evenness was highest in *C* (0.6), decreased in *T* (0.5), and was significantly lower in *F* (0.47) (*df* = 2, *F* = 9.9, *p* < .01; ANOVA), mainly due to the loss of diversity within Chlorophyta. The *T* treatment had the most significant effect on the number of genera richness (*p* < .0001, Tukey's HSD test), while species repartition was mainly influenced in *F* (*p* < .0001, Tukey's HSD test).

**Figure 5:**
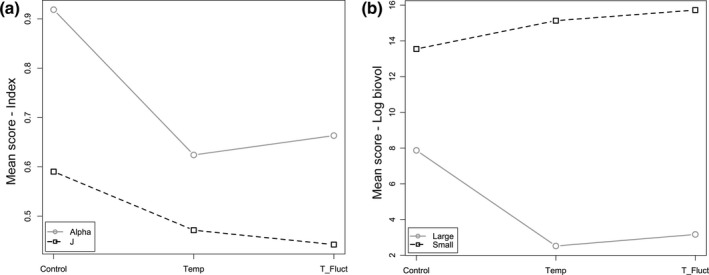
Profile analysis representing: (a) phytoplankton diversity (alpha index; gray circles) and evenness (J; black squares) among the control, temperature (Temp), and fluctuation (T_Fluct) treatments, and (b) phytoplankton biovolume size distribution (gray circles: large = 20–150 μm average cell length and black squares: small = 0–20 μm average cell length) during the duration of the entire experiment

The best‐fitting model for phytoplankton diversity included temperature and Δ_Temp as fixed variables and sampling time (Julian day) and mesocosm replicates as random effects. Model analysis of variance (ANOVA) revealed significant effects of temperature on the alpha index (*df* = 166, *F* = 4.1, *p* > .05), but not on the number of genera S (*df* = 166, *F* = 0.45, *p* > .05) and evenness J (*df* = 166, *F* = 0.1, *p* > .05). The strongest effect was evident from the Δ_Temp treatment on the number of genera (*df* = 166, *F* = 10.6, *p* = .001), on the alpha diversity (*df* = 166, *F* = 9.9, *p* = .001), and on the evenness (*df* = 166, *F* = 10.7, *p* = .001). The parallel analysis calculating the variation in the profile levels for the alpha index and the evenness (in Figure 5, the *x*‐axis presents the levels of the treatments and the *y*‐axis represents the calculated variance among the levels of the average diversity score) revealed a decreasing pattern for both indices, with the variables not overlapping among levels and significantly different from one another (*F* = 10.3, *p* = .001).

### 3.5 Community structure

**Figure 6:**
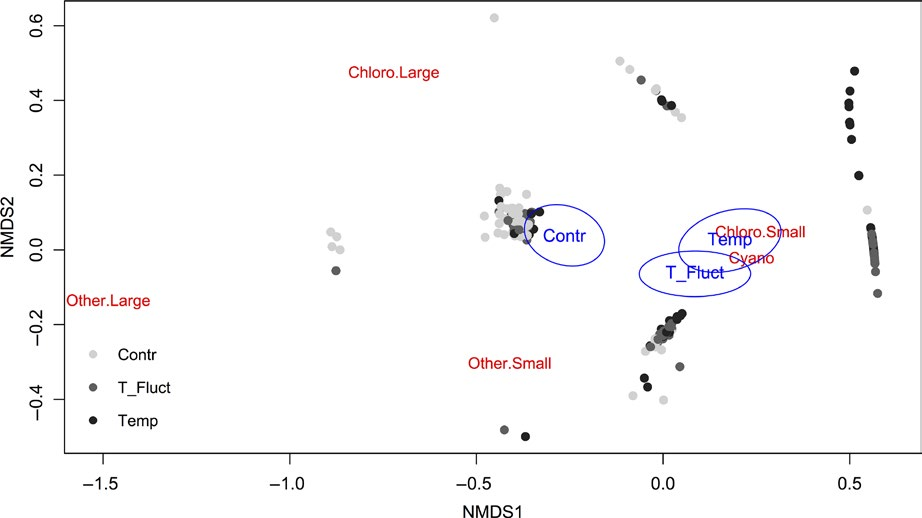
Phytoplankton biovolume NMDS. Max residuals 0.0001, nonmetric fit *R* ^2^ = 0.985, linear fit *R* ^2^ = 0.93, stress: 0.121, centroids confidence interval = 0.99. Chloro.Large = Chlorophyta >20 μm length, Chloro.Small = Chlorophyta <20 μm length, Other.Large = Euglenophyta, Dinophyta and diatoms >20 μm length, Other.Small = Euglenophyta, Dinophyta, and diatoms <20 μm length, Cyano = Cyanobacteria

The phytoplankton ordination (NMDS) clearly showed the distribution of the phytoplankton taxa among three centroids identified for the three treatments (Figure 6; max residuals 0.0001, nonmetric fit *R* ^2^ = 0.99, linear fit, *R* ^2^ = 0.98, stress: 0.123). The data cluster for *C* was identified by Desmids within the Chlorophyta and large algae represented from the other species (mainly Euglenophyta as *Trachelomonas* sp). The *T* and *F* treatments were associated with small Chlorophyta and Cyanobacteria. The environmental variability determined by the treatments (temperature and heat waves) significantly (*p* < .01) explained the variance in the phytoplankton distribution.

The best‐fitting model for small species biovolumes included temperature and TP as fixed variables and sampling time (Julian day) and mesocosm replicates as random effects. Model analysis of variance (ANOVA) revealed significant effects of temperature and TP (*df* = 166, *F* = 15.2, *p* = .0001, and *F* = 6.1, *p* = .01, respectively). For the Cyanobacteria biovolume, the best‐fitting model included temperature and Δ_Temp as fixed variable, with a significant effect (*df* = 167, *F* = 17.3, *p* = .01 and *F* = 4.05, *p* < .05, respectively).

Results from parallel analysis for the large and small phytoplankton species distribution revealed a variance pattern profile for large phytoplankton cells decreasing from the control (mean calculated score of biovolume log: 8) to the temperature and fluctuations treatments (mean scores between 2.5 and 3, respectively; Figure 5b). By contrast, the variance in small phytoplankton cells increased from the control to the treatments. The segments among level variables did not overlap and thus the profiles were significantly different from one another (*F* = 81.2, *p* < .001; Figure 5b).

The logistic regression analysis, run to investigate the effect of the treatments on the dominance of Cyanobacteria on the entire community, showed a significant effect of the treatments (*T*,* p* < .05; *F*,* p* < .001, Logit) driven by the temperature oscillations as environmental variable (*p* < .0001, ANOVA).

**Figure 7:**
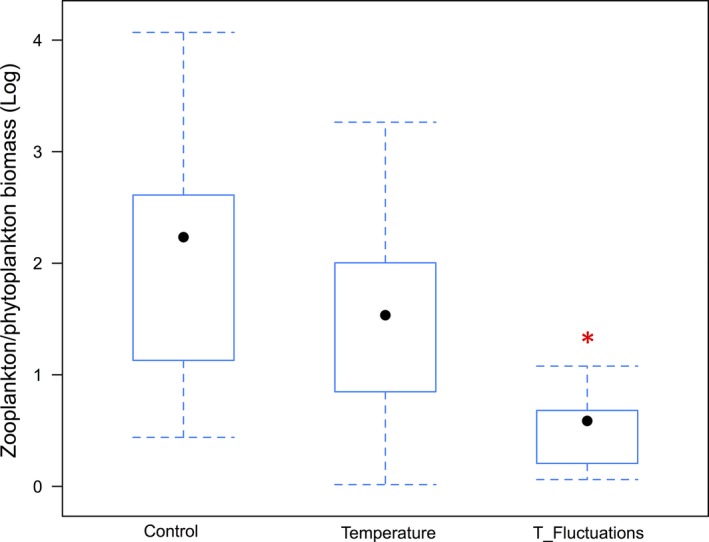
Zooplankton/phytoplankton biomass ratios in the three treatments averaged for the entire experiment. The box borders indicate the lower and upper quartiles, the dot in the center is the median, and the whiskers extending out from the box represent the maximum (up) and the minimum (down) of the data points. The red star represents significant differences (*p* = .001) for the *F* treatment (“*T*‐Fluctuations”) compared to the control and temperature treatment (ANOVA and Tukey's HSD test)

The zooplankton/phytoplankton biomass ratio (Figure 7) was higher in the control and lower in the temperature treatments with lowest ratio in the temperature fluctuation and significantly different form the control (*df* = 2, *F* = 7.2, *p* = .001; ANOVA).

## 4 Discussion

Temperature had the strongest effect in driving plankton abundance. During this 8‐month experiment, seasonal changes in plankton abundance were typical for middle latitudes with higher abundance during summer months and minimum in winter. As of February, phytoplankton and zooplankton started to increase their abundance and the response of the planktonic communities to the two temperature treatments, that is, *T* (constant) and *F* (pulse‐driven), were similar, and resulted in higher abundances compared to the control. This result underlines the importance of long‐term and multi‐seasonal experiments to reveal patterns and magnitude of the response to temperature changes that may not be detectable from typically performed short‐term experiments (Knapp et al., 2012) and account for changes that can vary seasonally (Kratina et al., 2012). We thus suggest that long‐term and multi‐seasonal mesocosm experiments are required to discern effects of changes on plankton communities, notably at middle latitudes where seasonal variability is also an important factor for plankton phenology (Sommer, Gliwicz, Lampert, & Duncan, 1986).

### 4.1 Plankton phenology

The significance of TP as driving factor for plankton abundances accounted for the importance of phosphorous supply for plankton growth, notably in oligo‐mesotrophic systems (Kalff & Knoechel, 1978) as our mesocosms. TP concentrations were higher in *T* and *F*, and as we introduced the same nutrient concentrations to all treatments, such higher TP concentrations may have been released from sediments due to higher temperatures (Jensen & Andersen, 1992). The effect of TP on heterotrophic bacteria in the *T* and *F* treatments is likely due to higher prokaryotes metabolic activities and nutrient uptake compared to the other planktonic population, notably at higher temperatures (Price & Sowers, 2004; Taucher & Oschlies, 2011). However, the minor importance of TP with respect to temperature as driving factor for plankton in the best‐fitting model corroborates the major effect of temperature and that the importance of nutrients was most likely temperature mediated. As the effect of temperature on bacterial activities also depends on the available resource pool (Hall, Neuhauser, & Cotner, 2008), we argue that warming promoted faster nutrient cycling, which thus favored the observed higher bacterial growth and heterotrophic bacteria abundance in *T* and *F* compared to *C*. However, the higher bacterial abundance in *T* did not change during the second part of the experiment, suggesting that consistently higher temperatures may account for a more stable community, better adjusted to the warmer environmental conditions compared to the pulse‐driven temperature changes in *F*.

Variations in phytoplankton abundance were also mostly affected by changes in temperature. This suggests that warmer temperatures have a direct effect on phytoplankton growth rate, which can likely be related to the sensitivity of the photosynthetic activity to temperature and the optimum temperature for the activation energy (Yvon‐Durocher et al., 2010). However, constantly higher temperatures in the *T* treatment caused higher algal abundance than in *F*, suggesting that these two temperature change scenarios (warmer and pulse‐driven temperatures) favored algal growth differently. A similarly high phytoplankton peak was recorded in our previous experiment (Rasconi et al., 2015), which confirms that increased temperature may trigger phytoplankton blooms once the community is adjusted to higher and rapidly fluctuating temperatures (Kosten et al., 2012), provided that nutrients are not limiting.

### 4.2 Phytoplankton community composition

The clear ordination of the phytoplankton community among three centroids identified for the three treatments confirmed our hypothesis that the induced environmental stressors pushed the community structure away from each other, with dominance of those species better adjusted to warmer and/or pulse‐driven temperatures. The *C* cluster was mainly characterized by large green algae species that typically proliferate during the summer planktonic successions in peri‐alpine, oligo‐mesotrophic lakes (e.g., *Cosmarium sp*., *Scenedesmus sp*. and *Oocystis sp*.) (Anneville, Gammeter, & Straile, 2005) and are also characteristic of nearby Lake Lunz (unpubl. data). Warmer temperature caused a clear shift toward a community more dominated by smaller species, including Chlorophyta and Cyanobacteria. Species proliferating in *T* at the beginning of the experiment were mainly Chlorophyta, but with time these were replaced by colonial Cyanobacteria (*Aphanoteche*,* Cyndrospermum* and *Oscillatoria*), together with small Chlorophyta, including *Chlorococcales ssp*. and colonial *Sphaerocystis*. In the *F* treatment, the main algal taxa were the same Cyanobacteria as in *T* together with the Chlorophyta *Cosmarium pygmaeum*. This shift in the size structure of the phytoplankton community toward smaller species can be driven by better adaptation of small species to warmer temperature (Bergmann, 1848) and faster growth rates (Lürling, Eshetu, Faassen, Kosten, & Huszar, 2013). Considering the important role of heterotrophic bacteria in nutrient cycling and higher nutrient availability, this shift from larger to smaller algae can also be due to better conditions related to a higher competitive ability of these cells in nutrient uptake (Banse, 1976; Litchman & Klausmeier, 2008). In the *F* treatment, the phytoplankton community composition shifted more toward a Cyanobacteria‐dominated community, supporting our hypothesis that unstable environmental conditions will push the pelagic food web to shift toward species more adjustable to rapid temperature change.

### 4.3 Diversity and dominance

The shift in phytoplankton community composition and structure was concomitant with a decrease of phytoplankton diversity genera richness in both temperature treatments compared to *C*. This was mostly due to loss of diversity within the Chlorophyta and the Dinophyta *Gymnodinium*, which was basically only detected in *C* during the second half of the experiment. The most important effect on diversity, however, was not on the decrease in the number of species detected in the treatment, but in their repartition, measured by the alpha diversity (measure of the local species pool among treatments) and evenness. Chlorophyta and Cyanobacteria were the most diversified groups in all treatments. However, as of February, while large desmids dominated in *C* (10.8% of relative biovolume) together with small Chlorophyta and Cyanobacteria (48.5% and 36.9% of relative biovolume, respectively), in *T* were dominant small Chlorophyta (51.2%) and Cyanobacteria (47.9%). In the *F* treatment, small Chlorophyta and Cyanobacteria represented about 99% of the relative biovolume, with 17.2% small Chlorophyta and 81.3% Cyanobacteria, which clearly indicates that these two temperature treatments induce the dominance of small‐celled algae and Cyanobacteria.

In lakes, progressive loss of phytoplankton diversity is often linked to a shift toward the dominance of Cyanobacteria (Kosten et al., 2012), which usually proliferate in warmer waters (Paerl & Huisman, 2008). In our experiment, Cyanobacteria were particularly abundant in F, in which also a strong bloom occurred during spring 2015, supporting the recent hypothesis that higher water temperatures promote Cyanobacteria dominance in shallow lakes (Kosten et al., 2012). The importance of temperature fluctuations and environmental instability for diversity loss and Cyanobacteria dominance was also supported by the calculated mixed model, which succeeded better in explaining Cyanobacteria growth and small‐celled species dominance when temperature oscillations were included. Indeed, studies conducted during one of the hottest recorded summers in Europe in 2003 showed that heat waves directly promoted Cyanobacteria blooms (Joehnk et al., 2008). This hypothesis was also supported by our logistic regression results, confirming that the *F* treatment significantly accounted for Cyanobacteria dominance. Taken together, our data suggest that consistently higher and pulse‐driven changes in temperature, as the case during reoccurring *F*, favored the abundance of Cyanobacteria.

The higher abundance of phytoplankton in *T* and the lower evenness is also attributable to the higher phosphorus availability in this treatment. Small Chlorophyta and Cyanobacteria are both considered fast‐growing r‐strategists that can endure higher temperature and favored in changing environments by their fast turnover, thus being able to develop blooms in very short period of time. In a laboratory experiment, Lürling et al., (2013) revealed that mean growth rates at the optimum temperature were similar for Cyanobacteria and Chlorophyta (0.92/day and 0.96/day, respectively, at 29.2°C). Moreover, due to the competitive advantage of Cyanobacteria to rapidly sequester nutrients, they are able to grow faster (r‐strategy) and outcompete other algae that are less efficient in nutrients uptake.

There was a clear effect of both temperature treatments (*T* and *F*) on phytoplankton size structure that resulted in significantly higher abundance of smaller species (i.e., 0–20 μm cell size: picoautotrophs and nanophytoplankton) than in the ambient‐temperature treatment (*C*). However, despite higher abundance of potentially more easily ingestible food the zooplankton/phytoplankton biomass ratio was lower in the *T* and *F* compared to *C*. This was due to the different phytoplankton composition driven by the two temperature treatments and notably the dominance of filamentous and colonial Cyanobacteria that are not readily edible and also constitute poor‐quality food for their consumers. The higher zooplankton/phytoplankton biomass ratio in the T treatment was due to the higher abundance of small Chlorophyta in this latter, which constitute better nutritional quality for consumer and thus trophic transfer was improved. However, the even higher zooplankton/phytoplankton biomass ratio in *C* suggests that higher phytoplankton diversity provided higher dietary quality for consumers and that a more diversified community is crucial to sustain a complex and diversified food web.

Decreased diversity and evenness in the *T* and *F* treatments pushed the community toward the dominance of only a few phytoplankton taxa that are better adapted to endure warmer and more irregular temperature conditions. However, despite the general high abundance of small phytoplankton species that are more readily ingestible for consumers, the trophic transfer was compromised in the warmer treatments. Notably in the *F*, the phytoplankton community was mainly dominated by colonial and filamentous Cyanobacteria, which are difficult to ingest by zooplankton (e.g., Lampert, 1987) and constitute poor‐quality food as it lacks, similar to other Cyanobacteria, sterols and omega‐3 polyunsaturated fatty acids that both support somatic growth and reproduction of zooplankton (Elert et al., 2003). We confirm thus our initial hypothesis and conclude: changes in temperature and reoccurring temperature fluctuations entailed an important diversity loss among the planktonic community and pushed the system toward Cyanobacteria dominance. This shift at the base of the food chain was reflected by lower consumer/producer biomass, which in turn might also cause changes in trophic energy transfer along the entire aquatic food web.

## Conflict of interest

None declared.
