# Supplementary material for: Temperature increase and fluctuation induce phytoplankton biodiversity loss – Evidence from a multi‐seasonal mesocosm experiment
Source: Ecol Evol. 2017 Mar 22;7(9):2936–46. doi: 10.1002/ece3.2889 (PMC5415537; doi:10.1002/ece3.2889)
Supplement: Supplementary file 1 [file ECE3-7-2936-s001.docx]

Supplementary data

Data S1: Seasonal variability in measured temperature for all mesocosms during the 8-months experiment (missing values are due to measurement problems of the system).

Supplementary figure

Fig. S1 Temperature variation in the temperature (T) and temperature fluctuation (F) treatments compared to the control.
