# Supplementary figures and images for: Temperature increase and fluctuation induce phytoplankton biodiversity loss – Evidence from a multi‐seasonal mesocosm experiment
Source: Ecol Evol. 2017 Mar 22;7(9):2936–46. doi: 10.1002/ece3.2889 (PMC5415537; doi:10.1002/ece3.2889)

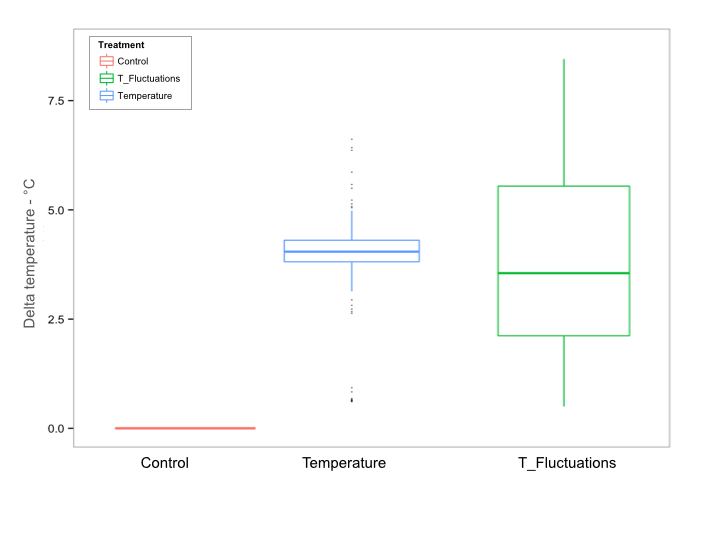

Supplement: Supplementary file 2 [file ECE3-7-2936-s002.jpg]
